# Emerging roles for microRNA in the regulation of *Drosophila* circadian clock

**DOI:** 10.1186/s12868-018-0401-8

**Published:** 2018-01-16

**Authors:** Yongbo Xue, Yong Zhang

**Affiliations:** Department of Biology, University of Nevada, Reno, 1664 North Virginia St., Reno, NV 89557-0315 USA

**Keywords:** Circadian clock, Drosophila, miRNA, Post-transcriptional regulation

## Abstract

**Background:**

The circadian clock, which operates within an approximately 24-h period, is closely linked to the survival and fitness of almost all living organisms. The circadian clock is generated through a negative transcription-translation feedback loop. microRNAs (miRNAs) are small non-coding RNAs comprised of approximately 22 nucleotides that post-transcriptionally regulate target mRNA by either inducing mRNA degradation or inhibiting translation.

**Results:**

In recent years, miRNAs have been found to play important roles in the regulation of the circadian clock, especially in *Drosophila*. In this review, we will use fruit flies as an example, and summarize the progress achieved in the study of miRNA-mediated clock regulation. Three main aspects of the circadian clock, namely, the free-running period, locomotion phase, and circadian amplitude, are discussed in detail in the context of how miRNAs are involved in these regulations. In addition, approaches regarding the discovery of circadian-related miRNAs and their targets are also discussed.

**Conclusions:**

Research in the last decade suggests that miRNA-mediated post-transcriptional regulation is crucial to the generation and maintenance of a robust circadian clock in animals. In flies, miRNAs are known to modulate circadian rhythmicity and the free-running period, as well as circadian outputs. Further characterization of miRNAs, especially in the circadian input, will be a vital step toward a more comprehensive understanding of the functions underlying miRNA-control of the circadian clock.

## Introduction

Circadian rhythms with approximately 24-h periods are prevalent in all living organisms, from unicellular cyanobacteria to vertebrates. This endogenous timekeeping system endows organisms with the enormous advantage of anticipating and adjusting to environmental changes (e.g., light, food, temperature, etc.) [1]. Many physiological and behavioral processes, including hormone secretion, thermoregulation, metabolism, immune responses, and sleep–wake cycles, are modulated by the circadian clock [2–5]. Therefore, it is not surprising that disruption of the circadian clock is associated with pathogenesis, such as obesity, cardiovascular diseases, and even cancer [6–8]. Since the circadian clock plays a fundamental role in the maintenance of circadian-dependent processes, understanding the regulatory mechanism of the circadian clock is vitally important.

**Fig. 1:**
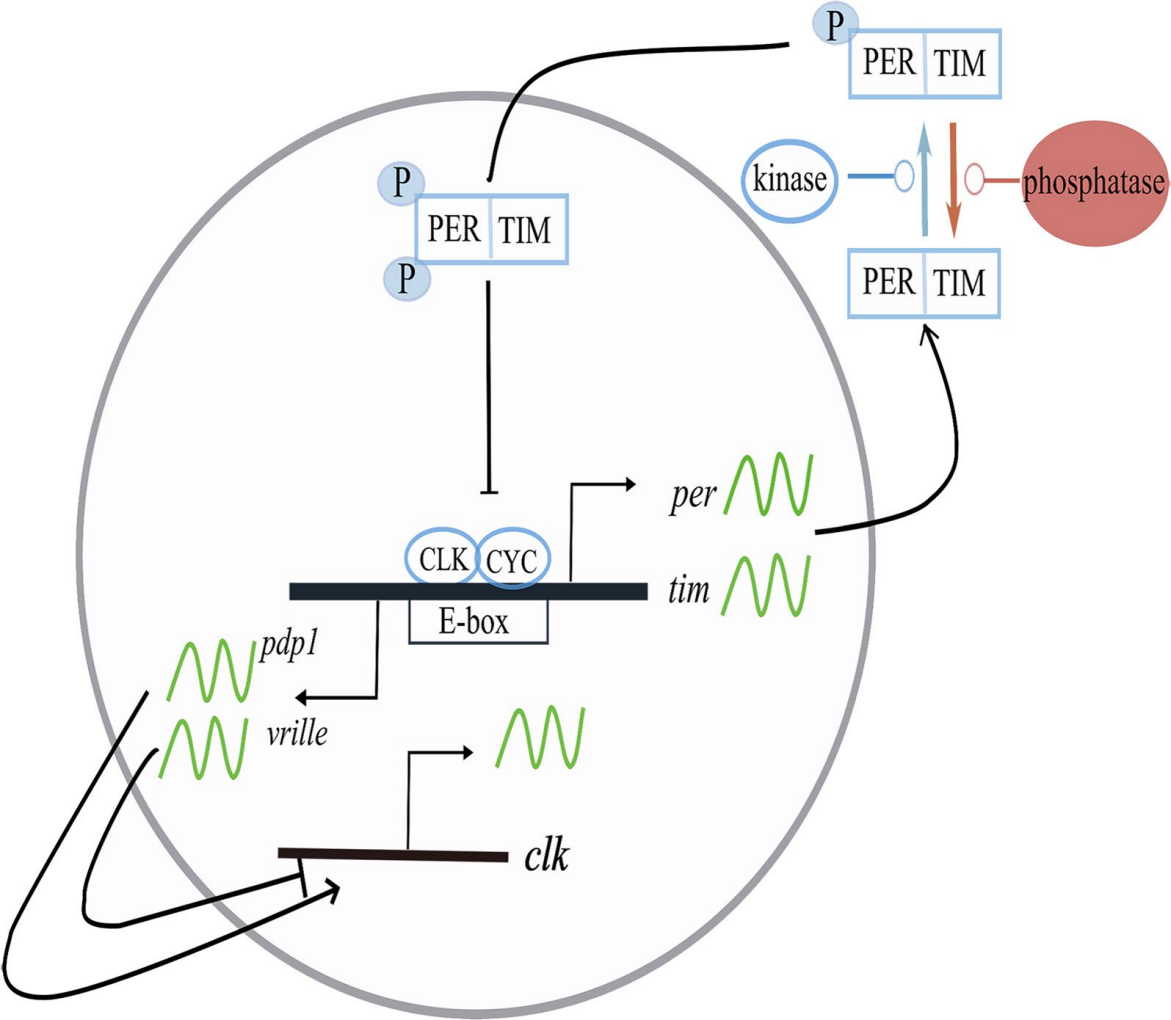
Circadian feedback regulatory loop in *Drosophila melanogaster*. In the *per/tim* loop, the CLK/CYC heterodimer binds to the E-box region of *per* and *tim* to activate their transcription. PER and TIM accumulate in the cytoplasm and form another complex at night. Phosphorylation of PER/TIM by kinases promotes their nuclear entry, which is counteracted by the dephosphorylation performed by phosphatases. The PER/TIM complex moves into the nucleus later in the night, and hyperphosphorylated PER/TIM represses CLK/CYC activity by promoting the phosphorylation of CLK. In the *clk/cyc* loop, CLK/CYC promotes the transcription of *pdp1* and *vri*, which encode PDP1 and VRI transcription factors to activate and repress *clk* transcription, respectively. VRI inhibition precedes PDP1 activation in a way that causes *clk* mRNA to oscillate during the day. CLK—CLOCK, CYC—CYCLE, PER—PERIOD, TIM—TIMELESS, pdp1—PAR domain protein 1, VRI—VRILLE

The clock’s machinery consists of exogenous, stimulus-sensitive input pathways, a central clock pacemaker capable of coordinating various endogenous pacemakers, and output pathways that produce overt rhythms [9]. The molecular components of the endogenous clock comprise a transcriptional-translational feedback loop (TTFL) that is fairly well-understood in mammals and flies. In the TTFL, the generation of rhythmic mRNA and protein abundance arises from repression- and activation-mediated temporal and spatial delays that eventually control downstream rhythmic behavioral patterns and physiological functions within an approximate 24-h period (Fig. 1).

*Drosophila* has been instrumental in the study of circadian rhythms because of powerful genetic tools and their relatively simple circadian neuronal circuitry. After more than four decades of studies in *Drosophila*, we now have a deep understanding of the molecular components of the circadian clock that play a pivotal role in coordinating circadian readouts to environmental inputs. Thus, in this review we will focus on the circadian rhythms of *Drosophila*.

In flies, approximately 150 circadian neurons make up the clock network in the brain. This network can be categorized into seven subclasses based on anatomical location, cellular size, and neuropeptide expression: the small and large ventral lateral neurons (LNvs) expressing pigment dispersing factor (PDF), the fifth small LNv, the dorsal lateral neurons (LNds), and three groups of dorsal neurons (DN1, DN2, and DN3) [10]. The core proteins of the TTFL, encoded by *clock* (*clk*), *cycle* (*cyc*), *period* (*per*), and *timeless* (*tim*), are expressed in all clock neuron groups, but transcriptome studies of individual circadian neurons have identified that most cycling transcripts are expressed only in a specific subgroup [11], which is likely due to different regulatory mechanisms. Moreover, next-generation sequencing (NGS) results have demonstrated that approximately 50% of rhythmic primary transcripts do not give rise to rhythmic mRNA, and rhythmic proteins do not necessarily arise from rhythmic mRNA transcripts [12, 13]. For these reasons, we sought to explore when, where and how post-transcriptional modification is integrated into the circadian control system. This review will focus on miRNA-dependent post-transcriptional regulation, due to accumulating evidence demonstrating the involvement of miRNAs in the regulation of circadian timekeeping.

**Fig. 2:**
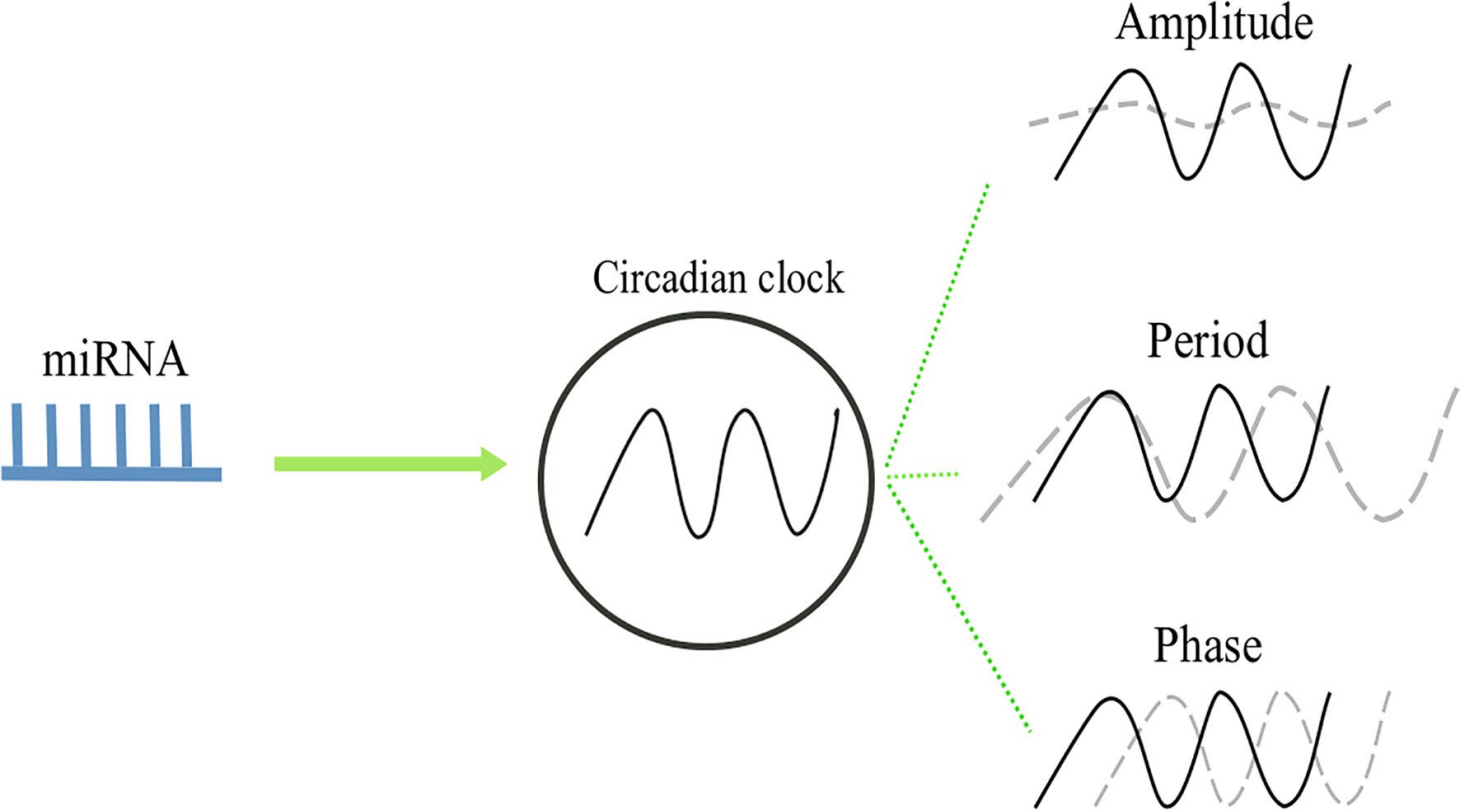
miRNA-mediated post-transcriptional regulation in different aspects of the circadian clock. Rhythmicity, period, and phase are important parameters of the circadian clock. Rhythmicity reflects the capability of flies to maintain the robust circadian clock; period reflects the speed of the circadian clock; and phase reflects the synchronization of the clock to the local environment. Solid black lines represent the oscillation of the clock. Dotted gray lines represent the miRNA-mediated corresponding changes to the clock

miRNAs are small non-coding RNAs of approximately 22 nucleotides (nt) that are generated by consecutive cleavage by the endoribonucleases, Drosha and Dicer. Drosha, an RNase III enzyme, recognizes primary miRNA (pri-mRNA) and cleaves it into an approximately 70-nt precursor miRNA (pre-miRNA) in the nucleus. Subsequently, pre-miRNA is exported into the cytoplasm and is subjected to Dicer-mediated cleavage that results in a dual miRNA products. One of the miRNA strands is then degraded and the other is loaded into the RNA-induced silencing complex (RISC). miRNA in RISC promotes degradation and attenuates translation by imperfect complementary binding to the non-coding 3′ untranslated region (UTR) of the target mRNA [14]. The most important determinant of mRNA expression in miRNA depends on the “seed sequence”, located at nucleotides 2–7 at the 5′ end of miRNA, which is used for the perfect complementary binding [15]. miRNAs regulate most physiological and biological pathways and processes [16], and circadian rhythms are no exception. In this review, we highlight the current knowledge of miRNA-mediated regulation of rhythmicity, the free-running period, and diurnal phase (Fig. 2). Furthermore, we also discuss approaches regarding the identification of circadian-relevant miRNAs.

## Transcriptional-translational feedback loops

The endogenous circadian clock maintains a free-running (in the absence of rhythmic environmental stimulus) period of approximately 24 h by employing interconnected feedback loops that typically include a primary loop and an ancillary loop. In flies, the primary negative feedback loop is composed of transcriptional activators (CLK/CYC) and repressors (PER/TIM) in which heterodimeric transactivators bind to promotor regions to induce repressor expression. Following 6–8 h of cytosolic repressor accumulation and modification, heterodimerization occurs, and the repressors translocate to the nucleus, inhibiting their own transcriptional activity by interacting with activators. Consequently, the levels of repressor complexes drop, and a new cycle of transactivation starts. The ancillary feedback loop shares core transactivators with the primary loop but differ in that Vrille (VRI) and Par domain protein 1 (PDP1) repress and activate *clk* transcription, respectively (Fig. 1). The ancillary feedback loop is believed to stabilize the molecular clock by regulating the phase and amplitude of core pacemakers and rhythmic outputs [17, 18].

In addition to the transcriptional control of the molecular clock, post-translational regulation is built into the TTFL to regulate protein stability, activity, and subcellular localization. Among different post-translational regulations, phosphorylation is one of the most interesting, as phosphorylation levels of principal clock proteins oscillate in a temporal pattern. For example, PER protein phosphorylation peaks in the early morning/late evening, and dPER is hypophosphorylated in the early evening [19]. The kinases involved in the phosphorylation of the PER/TIM complex are glycogen synthase kinase-3 [GSK3; also known as Shaggy (SGG)], casein kinase II (CK2), and DOUBLETIME (DBT; *Drosophila* homolog of mammalian CK1δ/ε) [20–23]. There are also several important phosphatases that dephosphorylate PER and TIM, such as Protein Phosphatase 1 (PP1) and Protein Phosphatase 2A (PP2A) [24, 25] (Fig. 1).

## Post-transcriptional regulation

The temporal delay between mRNA and protein oscillation of core clock genes indicates the existence of a layer of post-transcriptional regulation. When RNA sequencing and nascent RNA sequencing were applied to assess the whole genome circadian gene expression in fly heads, it was observed that ~ 77% strong, rhythmic, and mature mRNAs were derived from weak, non-oscillating nascent transcripts, suggesting that post-transcriptional modification has a major role in circadian mRNA expression^12^. This builds upon prior research that showed that only approximately 22% of cycling mRNAs exhibit phases identical to those of their de novo transcripts, and certain circadian-related genes were even found to display constant mRNA levels over the course of a day [26]. Together, these data strongly suggest that post-transcriptional regulation is essential for circadian gene expression.

Although post-transcriptional mechanisms, including alternative splicing, mRNA modification, poly(A) tail length, and alternative polyadenylation [27–30], are known to regulate cyclic gene expression, mRNA stability has been the main focus in the study of post-transcriptional regulation of circadian rhythms. Not surprisingly, mRNAs with short half-lives are more likely to display significant oscillations in expression than mRNAs with long half-lives are. For example, a handful of core clock genes in the TTFL, namely, *per*, *tim*, and *clk*, show mRNA oscillations because of variable half-lives, which are likely to be mediated by post-transcriptional regulation. Many studies have demonstrated that mRNA stability depends upon environment and on developmental stage [31], but the most crucial regulatory factors include *cis* elements in mRNA structure and trans-acting factors (RNA-binding proteins, RBP), such as LARK, for example, which is an RBP that is a key regulator of translation of *dbt* [32].

A recent study showed that deletion of the 3′UTR of *clk* mRNA in *Drosophila* led to ectopic expression of CLK, generation of additional PDF-expressing neurons, and the induction of variable circadian behavior [33]. In addition, deletion of binding sites in *clk* for the miRNA *bantam* resulted in a similar phenotype characterized by ectopic expression of VRI and overgrowth of PDF-positive neurons, suggesting that miRNAs regulate the development of circadian neurons [33].

## Roles of miRNA biogenesis in circadian regulation

Microarray-based experiments were used to identify miRNAs that show circadian oscillations in the fly head. Several conserved miRNAs, such as *miR*-*263a/b*, for instance, show strong daily oscillations [34]. Further analysis demonstrated that, in clock mutants, *miR*-*263a/b* have attenuated phases but elevated levels [34].

Later, studies demonstrated correlations between miRNA biogenesis and circadian rhythms. To monitor circadian behavior, flies are usually entrained by several 12-h light/12-h dark (LD) cycles and are then released into constant darkness (DD) for a few days. In DD, circadian rhythms are only dependent on the endogenous clock, as there are no environmental perturbations, and the entrained behaviors persist under DD. Downregulation of GW182, a protein that interacts with Argonaute (Ago) protein to modulate miRNA-mediated translational repression or mRNA degradation, in circadian neurons results in arrhythmicity under DD and an advanced evening phase with absent morning anticipation under LD [35]. The underlying mechanism for GW182 regulation of circadian rhythms appears to be downstream of the PDF-receptor signaling pathway [35].

*Dicer1*, which encodes an important ribonuclease gene for miRNA biogenesis, has also been implicated in the modulation of the circadian clock. Flies with downregulated Dicer1 in circadian neurons exhibited a lower amplitude of rest to activity rhythms [36]. This relatively weak effect on circadian rhythms might be due to the increased activity of repressors under these conditions, which likely buffers the translation from miRNA deficiencies [36]. The reduced amplitude is likely due to the downregulation of some circadian-relevant miRNAs. Interestingly, another study showed that the period of Dicer-deficient mice was shortened by approximately 2 h due to faster translation of two core clock proteins, namely, PER1 and PER2 [37].

## miRNA-mediated circadian amplitude

In the following sections, we will discuss several circadian-related miRNAs to illustrate the effects of specific miRNA regulatory mechanisms on circadian rhythms. The free-running circadian amplitude in DD reflects the self-sustainability of the circadian clock and serves as an important parameter for the evaluation of behavioral robustness. It has been shown that when some miRNAs are overexpressed, they severely impact the self-sustainability of the circadian clock. These miRNAs include *miR*-*279* and *miR*-*276a* [38, 39] (Fig. 2, Table 1).

**Table 1:** Characteristics of circadian-related miRNAs

miRNA	Target	Orcadian phenotype	Oscillation	Conservation
bantam	clk	Period	N	C. elegans and insects
let-7	cwo	Period and phase	Peak at ZT8 Trough at ZT16	C. elegans, Drosophila, Mouse, Humans
miR-279	upd	Rhythmicity	N	Insects
miR-276a	timeless	Rhythmicity	Peak at ZT10 Trough at ZT18	Insects
miR-124	–	Phase	Peak at ZT19 Trough at ZT7	C. elegans, Drosophila, Mouse, Humans
miR959-964	–	Immunity feeding	Peak at ZT12 Trough at ZT0	Drosophila
miR263a/b	–	–	Peak at ZT19 miR263a trough at ZT1 miR263b trough at ZT7	C. elegans, Drosophila, Mouse, Humans

Overexpression of *miR*-*279* in circadian neurons was found to cause arrhythmia in most flies [38]. Luo et al. [38] reported that *miR*-*279* regulated circadian rhythm output pathways by targeting the key component, *unpaired* (upd), in the JAK/STAT pathway. Though UPD is absent in the main pacemaker neurons (sLNv), it is prevalent in PER-positive neurons, DNs and laterally located neurons (LLNs), all of which are in close proximity to PDF-containing dorsal projections [38]. This further indicated that *miR*-*279* functions in circadian output. Notably, both overexpression and knockout of *miR*-*279* caused a high degree of arrhythmicity, possibly due to the multiple target effects or its essential role in the output pathway. Additional data have demonstrated that *miR*-*996* can rescue the behavioral phenotype in *miR*-*279* mutants [40]. *miR*-*996* is a circadian-relevant miRNA located downstream of *miR*-*279*, which shares a similar seed region. Overexpression of *miR*-*996* in circadian neurons was found to result in a phenotype similar to that of *miR*-*279* overexpression [40]. It seems that *miR*-*996* is redundant compared to *miR*-*279*, based on the regulatory capacities and similar seed region, as well as abundance, but the presence of *miR*-*996* may add to the robustness of *miR*-*279* regulation of circadian rhythms or may participate in unique functions.

Another miRNA known to influence the circadian clock is *miR*-*276a* [39]. *miR*-*276a* is positively regulated by the transcription factor Chorion factor 2 (CF2) and has a cyclic expression pattern under LD. Interestingly, flies overexpressing CF2 have a phenotype similar to that seen in flies with an overexpression of *miR*-*276a*. *miR*-*276a* appears to target the central clock gene, *tim* [39]. Analyses of flies with spatial and temporal misregulation of *miR*-*276a* showed that this miRNA is necessary for circadian behavior robustness in PDF-positive neurons (LNvs). TIM and PER levels were reduced following *miR*-*276a* overexpression. Reduced PER levels in PDF-positive neurons was deemed to be a secondary effect of direct targeting of the *tim* 3′UTR by *miR*-*276a*. Deletions of *miR*-*276a* binding sites in the *tim* 3′UTR phenocopied the deletion of *miR*-*276a* at both molecular and behavioral levels [39]. Interestingly, TIM overexpression did not fully rescue the arrhythmic phenotype. Two possible explanations for this observation are that the circadian clock may be extremely sensitivity to TIM dosage or that *miR*-*276a* targets additional circadian-relevant mRNAs.

## miRNA-mediated circadian period

The free running period is one of the most important characteristics of circadian rhythms as it reflects the speed of the circadian clock. To date, two miRNAs, namely, *bantam* and *let*-7, have been found to be involved in the regulation of the free-running period. These two miRNAs target two central clock genes, *clk* and *cwo*, respectively [36, 41] (Fig. 2, Table 1).

It is worth noting that *bantam* was the first miRNA found to affect the circadian rhythms of flies. The miRNA, *bantam*, was first shown to play vital roles in germline maintenance, peripheral nervous system dendrite growth, and eye development [42–44]. Through tiling arrays, Kadener et al. [36] found that disruption of miRNA processing in the nucleus resulted in the accumulation of *bantam* within fly heads, and overexpression of *bantam* in the main pacemaker neurons lengthens the circadian period. The molecular mechanism behind the circadian-period regulatory function of *bantam* involves interactions with three conserved target sites of the *clk* 3′UTR. An interaction between *clk* mRNA and RISC has also been shown by AGO1 immunoprecipitation [36]. It appears that *bantam* has other functions, as restoration of *clk* levels by expression of *clk* with a 3′UTR with no *bantam* binding sites did not rescue the arrhythmicity phenotype of *clk*^*AR*^.

Like *bantam*, *let*-*7* also modulates the circadian period in *Drosophila*. Along with two other non-redundant miRNAs, *miR*-*100* and *miR*-*125*, *let*-*7* is a co-transcriptional product from a single polycistronic locus, known as the let-7 complex (*let*-*7*-*C*) [45]. Previous studies of *let*-*7*-*C* miRNA were focused on its developmental functions. These miRNAs are important for the remodeling of the abdominal neuromusculature, ovary morphogenesis, nervous system formation, and lifespan control [45–48]. Expression of *let*-*7*-*C* is localized in the α/β lobes of the mushroom bodies [47], brain structures known to be essential for learning and memory regulation, as well as for sleep and locomotor activity [49, 50]. A recent study showed that *let*-*7* is also expressed in LNv neurons, further indicating the role of *let*-*7* in circadian rhythm regulation [41]. Abnormal expression of *let*-*7* in the main pacemaker neurons disrupts normal circadian rhythms: overexpression of *let*-*7* lengthens the period, whereas knockout of *let*-*7* eliminates morning anticipation [41]. Developmental defects were also excluded, as restricted overexpression in adulthood led to a long period of rest-activity rhythm [41]. Notably, *cwo*, which negatively regulates *clk* expression, was identified as the target of *let*-*7* both in vitro and in vivo [41]. Other phenotypes resulting from *let*-*7*-*C* knockout, such as abnormal PDF expression in PDF neuron projections and faster PER accumulation in LNv [41], may be due to secondary effects.

## miRNA-mediated phase determination in locomotion rhythms

Fruit flies exhibit a bimodal behavioral profile during each LD cycle, consisting of increased activity before dawn and dusk (termed morning anticipation and evening anticipation, respectively), both of which are controlled by dedicated circadian neurons [51]. Additionally, flies also display sharp and transient increases of activity during light–dark transitions, known as the “startle” response, which is a non-circadian response that persists even without a functional clock [51] or the main pacemakers [52]. *miR*-*124* is conserved across the animal kingdom and is abundantly expressed in the central nervous system [53] (Fig. 2, Table 1). In the fly head, *miR*-*124* expression is under circadian regulation, with trough and peak expression levels at mid-day and midnight, respectively [34]. Interestingly, *miR*-*124*-knockout flies show decreased morning anticipation under LD and an advanced evening phase under DD, but the circadian period is normal [54, 55]. In flies that do not express *miR*-*124*, circadian oscillators are functional with unaffected PER/TIM oscillation in all circadian neurons, and the photoreceptors show normal light response, suggesting that neither input or central clock functions are affected [54, 55]. Thus, *miR*-*124* may function in circadian output. Several potential targets of *miR*-*124* encode factors involved in the retrograde BMP signaling pathway, including Mef2, MMP1, and other positive components. However, expression of Mef2 and MMP1 and activation of BMP signaling regulate the endogenous period or rhythmicity under constant darkness [56–58], which is not consistent with the observed phenotype of *miR*-*124* mutants. A double heterozygous mutation of *sax* and *mad* was found to partially correct the phase shift in the *miR*-*124* mutant background [55], indicating the multifunction of *miR*-*124* in regulating circadian rhythms.

Unlike *miR*-*124*-mediated regulation of the locomotion phase, the *miR959*-*964* cluster mainly regulates the phase of feeding and immune function [59] (Table 1). The *miR959*-*964* cluster, which includes six miRNAs, is encoded by two introns proximal to the protein-coding gene, *CG31646* [59]. These six miRNAs show a robust circadian oscillation, which peaks at dusk (ZT12) [59]. The pri-miRNA cluster of *miR959*-*964* displays an even stronger oscillation than individual mature miRNAs do. This oscillation is abolished, and levels accumulate in the arrhythmic *per*^*01*^ mutant, and, oppositely, mature *miR959*-*964* levels are reduced in the *per*^*01*^ mutant. Surprisingly, expression pattern analysis did not localize expression of the *miR959*-*964* to circadian-relevant neurons, despite the fact that elimination of four of six miRNAs (*miR959*-*962*) displayed mild effects on the circadian period (approximately 0.5 h shorter), and overexpression of the cluster in TIM-positive neurons extended the period for more than 1 h [59]. In fact, this cluster is expressed in the peri-cerebral fat body (adult head fat body), an organ involved in metabolism and immunity [60], a finding that is consistent with the predication that most mRNA targets of *miR959*-*964* are implicated in metabolic and immune functions. The *miR959* ~ *964* cluster negatively modulates immune response, as the knockdown line exhibited dramatically lowered survival of the pathogen *Pseudomonas aeruginosa* [59]. The circadian-modulated feeding behavior was positively regulated by these six miRNAs, and there is a bidirectional regulation between feeding and levels of this cluster [59]. The circadian clock controls feeding time, and miRNAs in this cluster are likely to downregulate factors involved in responses to stress and starvation that regulate multiple downstream physiological processes, such as metabolism, feeding, and foraging time, until the stress is offset.

## Approaches to study circadian-relevant miRNAs

One direct approach that aims to identify circadian-relevant miRNAs involves monitoring circadian behaviors after performing a forward genetic screen, which entails manipulation of miRNA expression via knockout, overexpression, or downregulation. Recently, a large collection of miRNA mutants generated by targeted homologous recombination has become available [61]. These stocks enable genetic screening for circadian-relevant miRNAs. In addition, the Gal4/UAS system is a powerful tool for manipulating gene expression due to the availability and diversity of transgenic flies with UAS-miRNA or UAS-miRNA-sponge constructs [62]. Thus, by using tissue- and cell-specific drivers, it is feasible to manipulate functions of specific miRNAs only in certain organs. In addition, targeted spatial and temporal knockout of specific genes has become readily available with the emergence of the CRISPR/Cas9 system [63].

One efficient way to identify circadian-relevant miRNAs is to do RNA-sequencing (RNA-seq) in either all circadian or specific circadian neurons [64]. miRNAs can be extracted from specific circadian neurons after FACS isolation or by manual sorting [64]. The comprehensive and high-throughput nature of RNA-seq enables identification of relatively short, non-coding transcripts. Moreover, this approach provides great insights into the oscillation of mature miRNAs and the abundance of pri-miRNAs through intron mapping.

Characterization of miRNA targets is essential for understanding the mechanisms of how miRNA functions. Bioinformatic tools, such as TargetScan [65] and PicTar [66], have been widely used for predicting potential miRNA targets. In the future, in vitro studies can be performed by co-transfecting the miRNA and its predicted target gene 3′UTR with a reporter. If the miRNA binds to its target, a reduced reporter signal will be observed. Additionally, manipulation of target gene expression should phenocopy the effect of the miRNA in vivo. Finally, if a mutant mRNA that does not have miRNA binding sites in the target resembles the miRNA knockout phenotype, this would provide strong evidence supporting miRNA-target interactions.

## Concluding remarks

Emerging evidence has shown that miRNA-mediated post-transcriptional regulation is crucial to the generation and maintenance of a robust circadian clock in flies and mammals [9, 13, 26]. In this review, we discussed miRNAs known to modulate circadian rhythmicity and the free-running period, as well as locomotion and feeding phases in flies. Why is miRNA-mediated post-transcriptional regulation necessary in regulating the different attributes of circadian clock? One likely role is to contribute to the generation of the 24-h period. Although the established molecular mechanism of the circadian clock is based on the TTFL, it takes less than 24 h to close up the TTFL. Thus, additional regulatory levels, such as post-translational regulation and post-transcriptional regulation, for example, are required to generate the 24-h circadian period. A second likely role is to confer robustness to the running of the circadian clock. Multiple regulatory levels ensure that the circadian clock is less sensitive to changing environmental conditions. The third likely role is to enhance the mRNA oscillation of circadian-related genes as rhythmic mRNA expression is an important molecular output pathway of the circadian clock. Taken together, miRNA-mediated post-transcriptional regulation imposes benefits on the operation of the circadian clock.

As of yet, uncharacterized miRNAs may also play important roles in maintaining circadian rhythms. For example, miRNAs are found to be important for circadian photoresponses and seasonal adaptation in mammals [67], but no miRNA has been implicated in the circadian input pathways in flies yet. To date, 466 miRNAs have been identified in the fly genome (www.mirbase.org), but only a handful of miRNAs have been reported to be relevant to circadian clock function. Therefore, further characterization of miRNAs will be a vital step toward a more comprehensive understanding of the regulatory mechanisms underlying miRNA-mediated post-transcriptional control of the circadian clock.

## Abbreviations

AGO: Argonaute
CK2: casein kinase II
CLK: CLOCK
CWO: clockwork orange
CYC: CYCLE
DBT: doubletime
DD: dark: dark cycle
DN: dorsal neurons
GSK3: glycogen synthase kinase 3
LD: light: dark cycle
LNd: dorsal lateral neurons
LNv: ventral lateral neurons
miRNA: microRNA
PDF: pigment dispersing factor
PDP1: par domain protein 1
PER: PERIOD
PP1: protein phosphatase 1
PP2A: protein phosphatase 2A
RBP: RNA-binding proteins
RISC: RNA-induced silencing complex
TTFL: transcriptional-translational feedback loop
TIM: TIMELESS
UTR: untranslated region
VRI: VRILLE
